# Designing highly potent compounds using a chemical language model

**DOI:** 10.1038/s41598-023-34683-x

**Published:** 2023-05-07

**Authors:** Hengwei Chen, Jürgen Bajorath

**Affiliations:** Department of Life Science Informatics and Data Science, B-IT, LIMES Program Unit Chemical Biology and Medicinal Chemistry, Rheinische Friedrich-Wilhelms-Universität, Friedrich-Hirzebruch-Allee 5/6, 53115 Bonn, Germany

**Keywords:** Cheminformatics, Computational chemistry, Lead optimization

## Abstract

Compound potency prediction is a major task in medicinal chemistry and drug design. Inspired by the concept of activity cliffs (which encode large differences in potency between similar active compounds), we have devised a new methodology for predicting potent compounds from weakly potent input molecules. Therefore, a chemical language model was implemented consisting of a conditional transformer architecture for compound design guided by observed potency differences. The model was evaluated using a newly generated compound test system enabling a rigorous assessment of its performance. It was shown to predict known potent compounds from different activity classes not encountered during training. Moreover, the model was capable of creating highly potent compounds that were structurally distinct from input molecules. It also produced many novel candidate compounds not included in test sets. Taken together, the findings confirmed the ability of the new methodology to generate structurally diverse highly potent compounds.

## Introduction

Compound design is one of the major tasks for computational approaches in medicinal chemistry. The primary aim is the generation of compounds with desired properties, first and foremost, compounds with activity against individual pharmaceutical targets and high potency. For compound design and potency predictions, a variety of computational methods have been developed or adapted. Mainstays include quantitative structure–activity relationship (QSAR) analysis^1^ for the design of increasingly potent analogues of active compounds and methods for ligand- or structure-based virtual screening^2,3^ to identify new hits. Ligand- and structure-based methods have different requirements. For example, for docking calculations^4^, a variety of scoring functions have been developed to evaluate the quality and strength of receptor-ligand interactions and estimate binding energies^5,6^. For the structure-based prediction of relative potencies of congeneric compounds, free energy perturbation methods have been introduced^7,8^. At the ligand level, machine learning (ML) methods are widely used for hit identification and non-linear QSAR modeling^9^. For potency prediction, support vector regression (SVR)^10^ has become a standard ML approach. Furthermore, for both computational compound screening and potency prediction, deep neural network (DNN) architectures are also increasingly investigated^11–13^. Recently, a methodological framework was developed for evaluating the performance of deep generative models and a recurrent neural network (RNN) was used to explore predictions based on sparse training data^14^. However, the analysis mainly focused on physicochemical properties. For potency prediction, the assessment and comparison of different methods typically relies on the use of standard benchmark settings. Such benchmark calculations are required but not sufficient to evaluate potency prediction methods and their potential for practical applications. Moreover, such calculations should be considered with caution. Notably, in benchmark settings, nearest neighbor analysis and mean or median value regression often meet the accuracy of increasingly complex ML methods^15^. The high performance of these simple reference methods is supported by potency value distributions in commonly used compound data sets^15^. In addition, narrow error margins separating ML-based and randomized potency value predictions limit conclusions that can be drawn from conventional benchmarking^15^. Such findings call for alternatives to conventional benchmarking such as focusing predictions on the most potent data set compounds, consistent with the final goal of compound optimization efforts.

While potency predictions are mostly carried out for individual compounds, they can also be applied to assess potency differences in compound pairs such as activity cliffs (ACs), which are formed by structurally similar (analogous) active compounds with large differences in potency^16^. In principle, ACs can be predicted by explicitly calculating potency differences between compounds in pairs or by distinguishing between ACs and other pairs of analogues using classification methods, which implicitly accounts for potency differences of varying magnitude.

Previously, we have reported a deep learning approach for the prediction of ACs that further extended other ML classification methods by its ability to not only predict ACs, but also generate new AC compounds^17^. Since ACs encode large potency differences, we have reasoned that this methodology might be adapted and further extended for the design of highly potent compounds. Therefore, in this work, we have devised and implemented a chemical language model (CLM) for the prediction of highly potent compounds from weakly potent ones used as input. These predictions do not depend on conventional benchmark settings and are thus not affected by their intrinsic limitations.

## Methods

### Compounds, activity data, and analogue series

From ChEMBL (release 29)^18^, bioactive compounds with high-confidence activity data were assembled. Only compounds with reported direct interactions (assay relationship type: “D”) with human targets at the highest assay confidence level (assay confidence score 9) were considered. As potency measurements, only numerically specified equilibrium constants (K_i_ values) were accepted and recorded as (negative logarithmic) pK_i_ values. If multiple measurements were available for the same compound, the geometric mean was calculated as the final potency annotation, provided all values fell within the same order of magnitude; otherwise, the compound was disregarded. Qualifying compounds were organized into target-based activity classes. A total of 496 activity classes were obtained.

For each activity class, a systematic search for analogue series (ASs) was conducted using the compound-core relationship (CCR) method^19^, which uses a modified matched molecular pair (MMP) fragmentation procedure^20^ based on retrosynthetic rules^21^ to systematically identify ASs with single or multiple (maximally five) substitution sites. The core structure of an AS was required to consist of at least twice the number of non-hydrogen atoms of the combined substituents^19^.

**Figure 1:**
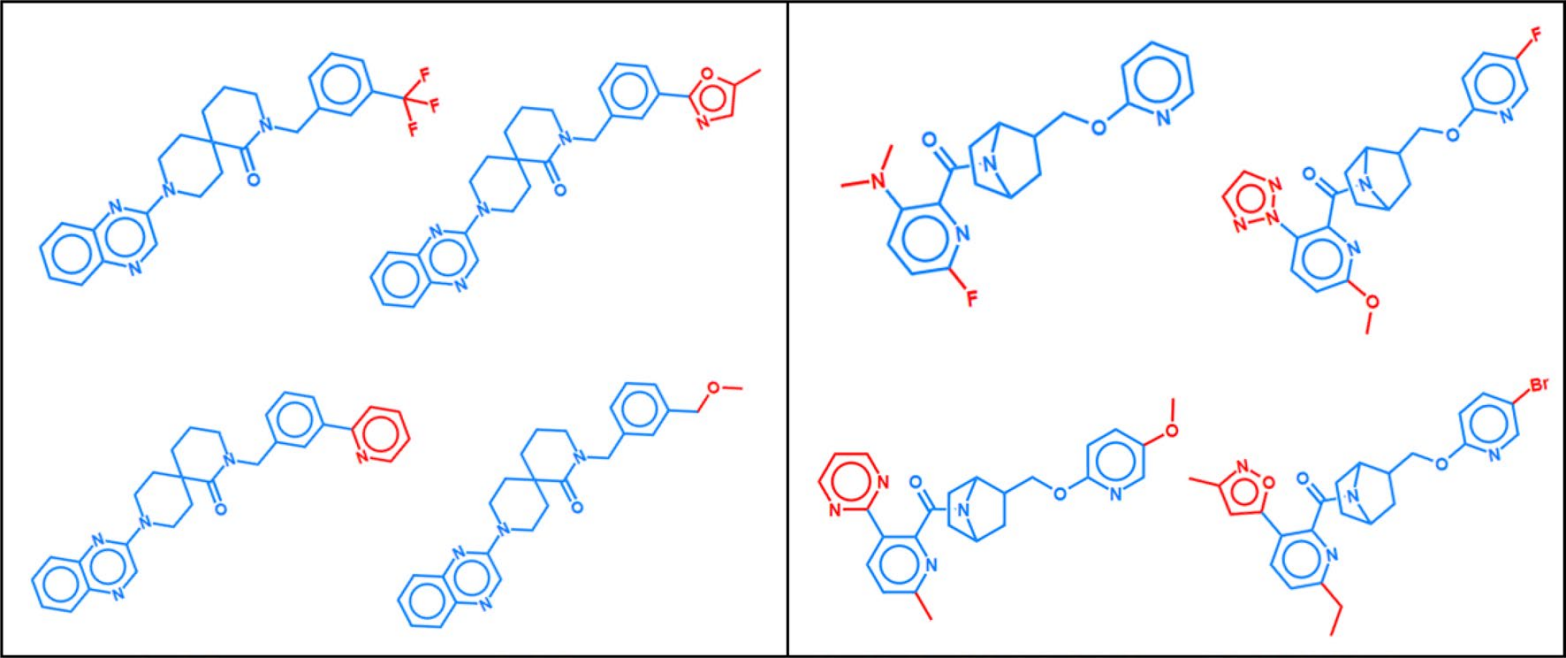
Exemplary analogue series. Shown are small ASs with single (left) or multiple substitution sites (right). Core structures are colored blue and substituents red.

Ultimately, 10 classes comprising ligands of different G protein coupled receptors were extracted as test cases for compound predictions that each contained more than 900 compounds and more than 100 analogue series. Table 1 summarizes the targets and composition of these activity classes (first four columns from the left) and Fig. 1 shows exemplary ASs with single or multiple substitution sites.

**Table 1:** Activity classes.

ChEMBL ID	Target name	Compounds	ASs	CCR pairs	AC-CCR pairs
218	Cannabinoid CB1 receptor	1118	250	8889	585
226	Adenosine A1 receptor	1924	318	18,623	1207
233	Mu opioid receptor	1216	169	10,430	1110
234	Dopamine D3 receptor	1529	213	21,008	755
237	Kappa opioid receptor	940	129	19,277	2897
251	Adenosine A2a receptor	1825	312	16,084	870
256	Adenosine A3 receptor	2033	434	42,621	6219
3371	Serotonin 6 receptor	1535	201	36,735	2485
4792	Orexin receptor 2	1133	131	12,368	1271
5113	Orexin receptor 1	1086	155	23,169	817

For each of 10 activity classes, the number of compounds, ASs, CCR pairs, and AC-CCR pairs are provided. In addition, for each class, the ChEMBL target ID, target name, and abbreviation are given. AS, CCR, and AC stand for analogue series, compound-core relationship, and activity cliff, respectively.

**Figure 2:**
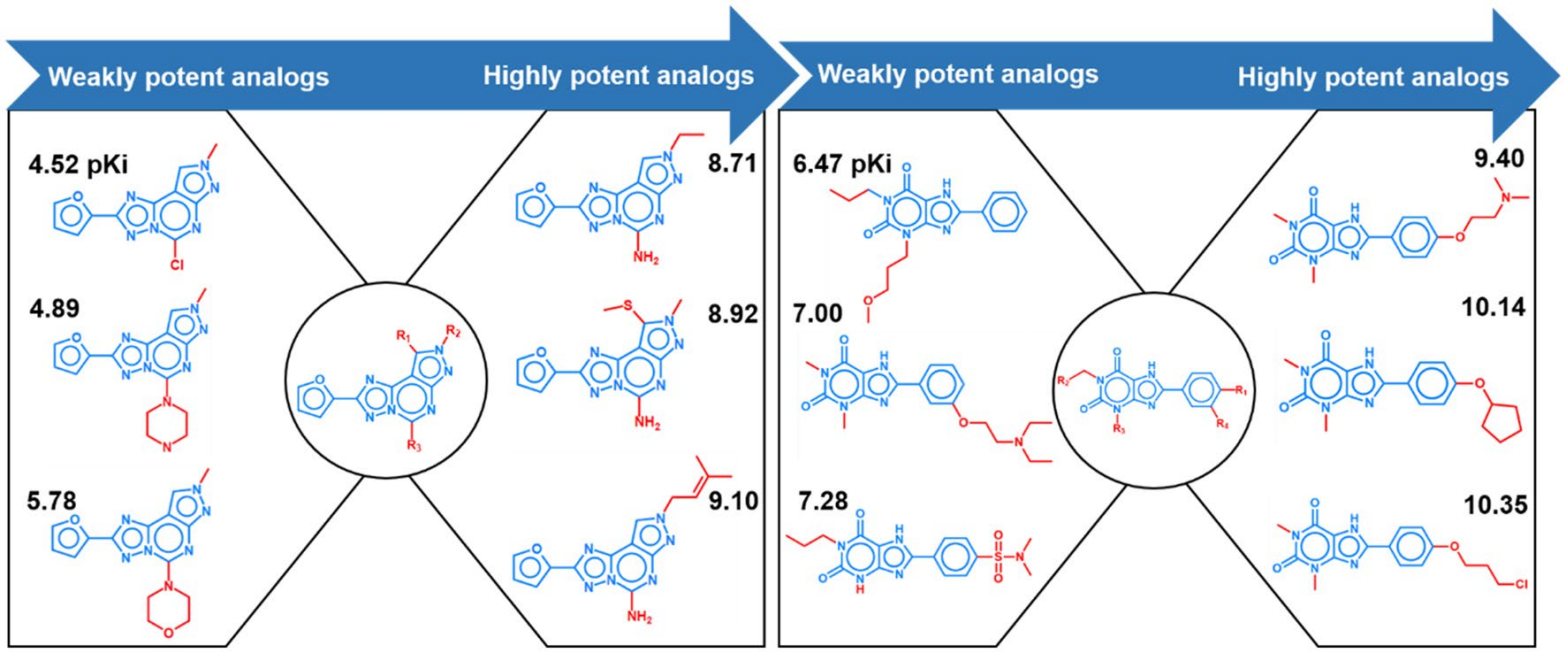
Analogue pairs. For each of two exemplary ASs, three representative All_CCR pairs are shown (top, middle, and bottom; increasing potency from the left to the right). The Markush structure representing each AS is displayed in the center. Core structures are colored blue and substituents red. For each compound, its pK_i_ value is reported.

From each of the activity classes, all possible pairs of analogues (termed *All_CCR* pairs) were extracted, as illustrated in Fig. 2 that shows All_CCR pairs for two different ASs. The 496 activity classes yielded a total of 881,990 All_CCR pairs.

#### Tokenization

For use by a CLM, compounds and potency differences must be tokenized. All compounds were represented as molecular-input line-entry system (SMILES) strings^22^ generated using RDKit^23^ and tokenized using a single chemical character with the exception of two-character tokens (i.e., “Cl” and “Br”) and tokens in brackets (e.g. “[nH]” and “[O-]”). For the conditional transformer, potency differences must also be transformed into input tokens. For tokenization of value ranges, different approaches have been introduced including binning^17,24,25^ and, more recently, numerical tokenization^26^. Since human readability of token sequences supported by numerical approaches played no role for our analysis and encoding of drug discovery-relevant compound potency ranges via binning has yielded accurate predictions previously^17^, we continued to use binned tokens herein. Accordingly, potency differences between source and target compounds, ranging from − 6.62 to 6.52 pK_i_ units, were partitioned into 1314 binned tokens of a constant width of 0.01. This granularity (resolution) defines the limits of experimental potency measurements and was thus most appropriate for our analysis. Each bin was encoded by a single token and each potency difference was assigned to the token of the corresponding bin^17^.

Tokenization of compound SMILES strings and potency ranges yielded the chemical vocabulary for our model. In addition, the two special tokens “start” and “end” were added to the vocabulary indicating the start and end point of a sequence, respectively.

### Generative chemical language model

#### Architecture

For compound design, a CLM with the transformer architecture previously reported for the DeepAC approach for AC prediction^17^ was used. The transformer architecture consisted of multiple encoder-decoder neural modules with attention mechanism^27^. In the model, a stack of encoding sub-layers including a multi-head self-attention sub-layer and a fully connected feed-forward network sub-layer constituted the encoder module. The encoder read an input sequence and compressed it into a context vector in its final hidden state. The context vector served as the input for the decoder block that interpreted the vector to predict an output sequence. Subsequently, the decoder module, which was composed of a feed-forward sub-layer and two multi-head attention sub-layers, re-converted the encodings into a sequence of tokens (one token at a time). Both encoder and decoder utilized the attention mechanism during training to comprehensively learn from feature space.

During pre-training, the model was supposed to learn mappings of source to target compounds based on potency differences resulting from changes in substituent(s) (termed chemical transformations):

$$(Source\;compound,\;Potency\;difference) \to \left( {Target\;compound} \right).$$

Then, given a new *(Source compound, Potency difference)* test instance, the model was applied to generate a set of candidate target compounds meeting the potency difference constraints, that is, having higher potency than the source compound (according to the given potency difference).

During pre-training, distinguishing between different activity classes was not required because at this stage, the model should learn the syntax of textual molecular representations and, in addition, a variety of analogue pair-associated potency differences caused by chemical transformations. By contrast, during fine-tuning, activity class (target) information was required to focus the model on specific compound series or classes, as further discussed below.

#### Model derivation

The transformer model was implemented using Pytorch^28^. Default hyperparameter settings were used together with a batch size of 64, learning rate of 0.0001, and encoding dimension of 256. The models were derived over 200 epochs on the basis of the general training set. During training, the transformer model minimized the cross-entropy loss between the ground-truth and output sequence. A checkpoint was saved at each epoch and for a validation set, minimal loss was determined for selecting the final model.

### Model pre-training

A general data set for model pre-training was derived from the 881,990 All_CCR pairs of the 496 activity classes. From All_CCR pairs, All_CCR triples *(Cpd*_*A*_*, Cpd*_*B*_*, Pot*_*B*_*-Pot*_*A*_*)* were generated by recording the potency difference for an All_CCR pair. Here, *Cpd*_*A*_ represented the *source compound* that was concatenated with the potency difference (*Pot*_*B*_*-Pot*_*A*_) and *Cpd*_*B*_ represented the *target compound*. For each All_CCR pair, two triples were obtained such that each All_CCR compound was used once as the source and target compound. To avoid data ambiguities, All_CCR pairs were eliminated if (1) a given source compound and potency difference was associated with multiple target compounds from different activity classes or (2) multiple potency values from different classes were available for a pair. On the basis of these criteria, a curated general data set of 522,331 qualifying All_CCR triples was obtained and used for pre-training.

For each triple, the SMILES representation of the source compound concatenated with the binned token of the associated potency difference served as the input sequence for the encoder that was converted into a latent representation. Based on this representation, the decoder iteratively generated output SMILES sequences until the end token was detected.

### Model fine-tuning

For model fine-tuning and evaluation, the 10 activity classes in Table 1 were used. For fine-tuning, All_CCR pairs were extracted from each of the 10 activity classes and divided into subsets of so-called *CCR* pairs with a less than 100-fold potency difference and *AC-CCR* pairs capturing an at least 100-fold difference in potency. Accordingly, AC-CCR pairs represented analogue pairs forming ACs. Depending on the activity class, 8889–42,621 CCR pairs and 585–6219 AC-CCR pairs were obtained (Table 1, last two columns on the right). AC-CCR triples were ordered such that *Cpd*_*B*_ was highly and *Cpd*_*A*_ weakly potent.

The pre-trained model was then separately fine-tuned and tested for each activity class. Therefore, AC-CCR pairs from each class were randomly divided into 80% fine-tuning and 20% test instances such that there was no overlap in core structures between these sets. Thus, the fine-tuning set exclusively consisted of AC-CCR pairs and was selected to train the model on activity class dependent analogue pairs with large potency differences. CCR pairs sharing core structures with the fine-tuning set were omitted from further consideration. The remaining CCR pairs were added to the test set. Hence, the fine-tuning and test sets were structurally distinct. Model evaluation is detailed below.

## Results

### Study concept

Our study had three primary goals. First, we aimed to devise a novel approach specifically for predicting highly potent compounds from weakly potent input molecules. Thus, rather than striving for prediction of potency values across large ranges, as is conventionally attempted using SVR or other machine learning methods, the primary focus was on potent compounds, in line with the practical relevance of potency predictions. Second, we aimed to generate a structural spectrum of output compounds, ranging from analogues of input molecules to structurally distinct compounds, thereby increasing medicinal chemistry novelty of predicted candidates. Third, it was intended to evaluate the methodology in a way that was not affected by limitations of conventional benchmarking of potency predictions, as discussed above, and enabled a non-ambiguous assessment of the ability to predict potent compounds. To meet the first two goals, which were central to our study, we implemented a CLM consisting of a chemical transformer architecture conditioned on compound potency differences. To meet the third goal, we designed a new compound test system.

### Compound pair-based test system

For model evaluation, a compound pair-based test system was generated using the test set. By design, the fine-tuning and test sets were structurally distinct. Furthermore, in contrast to the fine-tuning set, the test set contained analogue pairs capturing small or large differences in potency (i.e., CCR and AC-CCR pairs, respectively). Table 2 summarizes the composition of the test set.

**Table 2:** Test set.

ChEMBL ID	CCR pairs	Unique CCR CPDs	AC-CCR pairs	Unique AC-CCR CPDs	Overlapping CPDs	Unique CCR + AC-CCR CPDs	SCs (pki ≤ 6)	KCCs (pki > 6)
218	2198	579	6	12	9	582	129	453
226	5950	1174	144	84	80	1178	359	819
233	2332	590	36	36	33	593	76	517
234	7790	913	50	53	53	913	89	824
237	1032	477	31	24	20	481	115	366
251	4706	5210	85	57	38	5229	1391	3838
256	5012	888	40	44	42	890	250	640
3371	4420	722	42	44	44	722	40	682
4792	1941	615	49	50	48	617	146	471
5113	7543	664	13	15	15	664	256	408

CPD stands for compound, SC for source compound, and KCC for known candidate compound. According to our analysis scheme, target compounds (TCs) produced by the model were compared to KCCs.

For each activity class, the test set contained varying numbers of CCR pairs and AC-CCR pairs yielding varying numbers of unique CCR and AC-CCR compounds. In the following, *SC* and *TC* are used as abbreviations for source (input) and target compound, respectively. For the evaluation of the fine-tuned CLM, test set compounds were divided into instances with maximally 1 μmol potency (corresponding to a pK_i_ value of 6), which served as SCs, and candidate compounds with higher than 1 μmol potency (pK_i_ > 6), which served as *known candidate compounds* (*KCCs*) for comparison with newly generated TCs.

In addition, the model generated varying numbers of novel (hypothetical) TCs. For each activity class, smaller numbers of SCs than KCCs were available. With the exception of activity class 251 (3838 KCCs), the test set contained 366–824 KCCs for the activity classes (Table 2), with on average 576 KCCs per class. Each CCR-SC (pK_i_ ≤ 6) and AC-CCR-SC (pK_i_ ≤ 6) was once used as an input compound for the model and in each case, 50 TCs were sampled, canonicalized, and compared to KCCs to search for exact matches, that is, fully reproduced compounds with known potency. Because the model generated novel TCs, probabilities for re-generating known TCs could not be derived in a meaningful way. Consequently, the main measure for establishing proof-of-principle for the ability of the model to predict potent compounds was the reproduction of *any KCCs*. For each activity class, compound statistics were derived over three independent sampling trials, as reported below.

Table 3 reports the possible predictions outcomes for the compound pair-based test system.

**Table 3:** Possible predictions.

Index same/different core	Compound pair category
1.1./1.2.	(CCR-SC, CCR-TC)
1.3./1.4.	(CCR-SC, AC-CCR-TC)
1.5./1.6.	(CCR-SC, novel CPD)
2.1./2.2.	(AC-CCR-SC, AC-CCR-TC)
2.3./2.4.	(AC-CCR-SC, CCR-TC)
2.5./2.6.	(AC-CCR-SC, novel CPD)

For each SC, a TC could be a known CCR or AC-CCR compound or a novel (hypothetical) compound representing a TC not contained in the fine-tuning or test set. Taking core structure matches into consideration (that is, a TC either contained the same core structure as a SC or not), a total of 12 formally defined prediction outcomes were possible, including six each for CCR-SCs and AC-CCR-SCs, as identified by indices 1.1.–1.6. and 2.1.–2.6. in Table 3, respectively. Accordingly, a newly generated compound might be a structural analogue of a given SC (having the same core structure) or contain a different core structure. Furthermore, SCs and TCs might be distinguished by single or multiple substituents. On the basis of this classification scheme, CLM predictions were rigorously evaluated focusing on the reproduction of known active compounds, as explained above. This was the most relevant measure of model performance because it enabled the exact determination of potency differences between SCs and TCs and hence the ability of the CLM to predict highly potent compounds. For novel (hypothetical) compounds generated by the model, no assessment was possible (without subsequent experimental evaluation).

### Model performance

For the SCs from all activity classes, systematic compound predictions were carried out using the CLM. The model only produced 0.5–2% invalid SMILES (assessed using RDKit) for all activity classes.

With the exception of class 251 (1391 SCs), the test set contained 40–359 SCs for the activity classes, with on average 162 compounds per class (Table 2). The predictions were then assessed on the basis of well-defined pair categories detailed above, as reported in Table 4.

**Table 4:** Prediction results.

ChEMBL ID	1.1.	1.2.	1.3.	1.4.	1.5.	1.6.	2.1.	2.2.	2.3.	2.4.	2.5.	2.6.
218	73	192	2	5	436	3301	3	3	4	11	24	34
226	262	433	4	25	1067	5030	11	11	27	79	129	529
233	217	179	2	14	252	570	6	9	0	10	21	45
234	141	92	3	2	286	705	3	2	6	7	24	13
237	488	250	0	11	181	766	9	26	14	4	4	10
251	2367	1400	235	128	1031	13,523	17	5	36	13	55	199
256	112	66	1	2	657	5336	10	7	0	12	13	359
3371	60	116	0	4	42	1202	7	4	3	8	33	101
4792	224	662	7	42	253	1222	7	6	7	17	17	25
5113	433	349	1	5	304	1638	5	2	11	2	15	24

For each activity class and compound pair category indexed according to Table 3 (top row), the number of unique TCs produced by the CLM is reported. With the exception of categories 1.5., 1.6., 2.5., and 2.6., which report novel (hypothetical) candidate compounds not contained in the fine-tuning or test set, the TCs represent KCCs, as defined in the text.

Encouragingly, for all activity classes, the CLM successfully reproduced large numbers of KCCs for all SCs (categories 1.1.–1.4. and 2.1.–2.4., respectively). Frequently, multiple KCCs were obtained for the same SC. Furthermore, depending on the activity class, the model produced varying numbers of TCs with the same or different core structure, thus confirming its ability to generate frequent core structure transformations. In many cases, more structurally unique TCs were generated than analogues of SCs. Moreover, large numbers of hypothetical candidate compounds not contained in the training set were obtained (categories 1.5.–1.6. and 2.5.–2.6., respectively). The reproducibility of the limited numbers of available KCCs representing known ACs (12–84 unique compounds per activity class) was of particular interest (categories 2.1.–2.4.). AC-CCR KCCs were consistently reproduced and for five activity classes, the total count exceeded the number of unique AC-CCR KCCs per class (due to multiple reproductions of individual KCCs). Table 5 reports statistics for reproduction of KCCs.

**Table 5:** Reproducibility of *known candidate compounds*.

ChEMBL ID	KCCs	Non-KCCs	Reproduced KCCs (%)
218	103	3445	22.74 ± 1.10
226	211	5139	25.76 ± 0.49
233	143	1005	27.66 ± 1.35
234	92	825	11.17 ± 0.24
237	128	839	34.97 ± 1.37
251	251	4996	6.54 ± 0.29
256	76	5165	11.88 ± 0.63
3371	72	2145	10.56 ± 1.17
4792	172	1084	36.52 ± 1.91
5113	117	1499	28.68 ± 1.72

Reported are statistics for the re-generation of KCCs including the mean number of KCCs over three independent sampling trials and the proportion of reproduced KCCs relative to all available KCCs with standard deviations (±). In addition, the mean number of non-KCCs over three independent trials is provided.

The proportion of exactly reproduced KCCs over independent sampling trials ranged from ~ 7 to ~ 37%, depending on the activity class (with generally small standard deviations). For nine, six, and two classes, more than 10, 20, and 30% of all available KCCs were reproduced, respectively. Applying the most rigorous criterion of exact re-generation of known potent compounds as a performance measure (see above), the observed numbers and proportions represented unexpectedly good predictions, which clearly established proof-of-concept for the approach.

For each activity class, ASs were also extracted from newly generated (predicted) compounds. Table 6 reports the number of ASs (multiple compounds having the same core structure) and singletons (compounds with unique core structures not belonging to any AS). Depending on the activity class, 90–1414 ASs and 219–1762 singletons were obtained, respectively.

**Table 6:** Structural organization of predicted compounds.

ChEMBL ID	ASs	Singletons	Reproduced cores (%)
218	858	1235	4
226	905	1762	4
233	188	255	12
234	90	245	9
237	175	303	7
251	1304	978	4
256	1414	1386	7
3371	321	1022	4
4792	146	219	18
5113	233	440	9

“Reproduced cores” reports the percentage of the core structures contained in each original activity class that were detected in predicted compounds.

Since each AS and singleton contained a unique core structure (scaffold), the core structure diversity of newly generated compounds was generally high. Between 4 and 18% of the core structures contained in the original activity classes (from ASs and singletons) were reproduced by the model, as also reported in Table 6.

**Figure 3:**
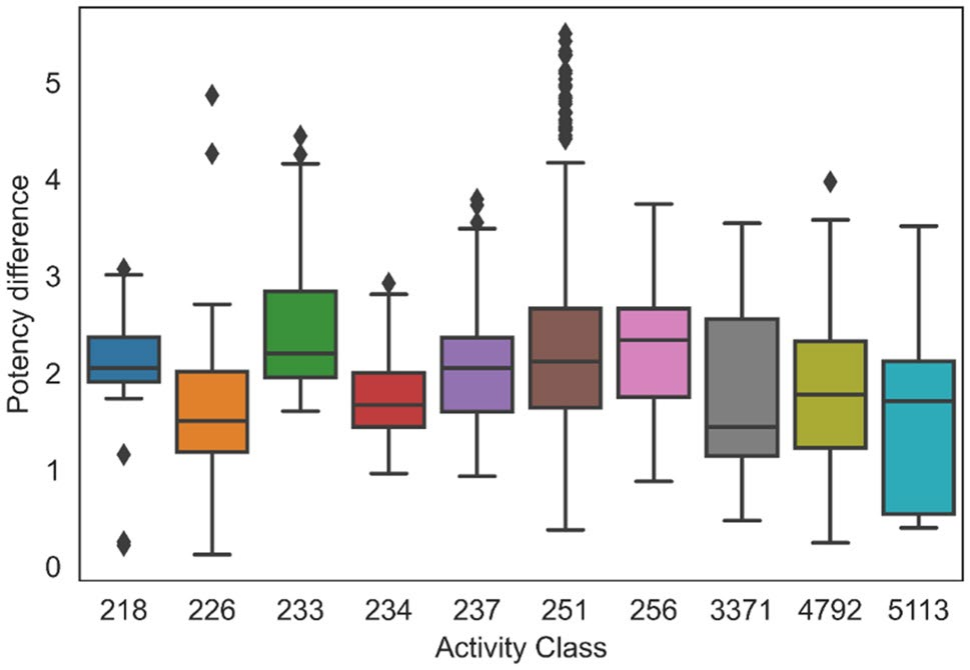
Potency difference distribution. For all activity classes, boxplots report the distributions of logarithmic potency differences between pairs of known source and target compounds involving compounds from ACs. In boxplots, the median value is represented by the horizontal line, and the box defines upper and lower quantile. Upper and lower whiskers represent the maximum and minimum value, respectively. Diamond symbols mark statistical outliers.

Having confirmed the ability of the CLM to generate structurally analogous and diverse TCs including KCCs, the key question then was whether or not the model would produce TCs that had much higher potency than the corresponding SCs. Figure 3 shows the distributions of potency differences between pairs of known source and target compounds with experimental potency values involving compounds from ACs. For five activity classes, the median potency difference fell between one and two orders of magnitude (10–100-fold) and for the other five classes, the median value exceeded two orders of magnitude (100-fold). Furthermore, for all but one class, multiple compounds with at least 1000-fold higher potency than the corresponding SCs were generated (including highly potent statistical outliers). Thus, these observations unambiguously confirmed the ability of the CLM to generate highly potent compounds from weakly potent (micromolar) input molecules.

**Figure 4:**
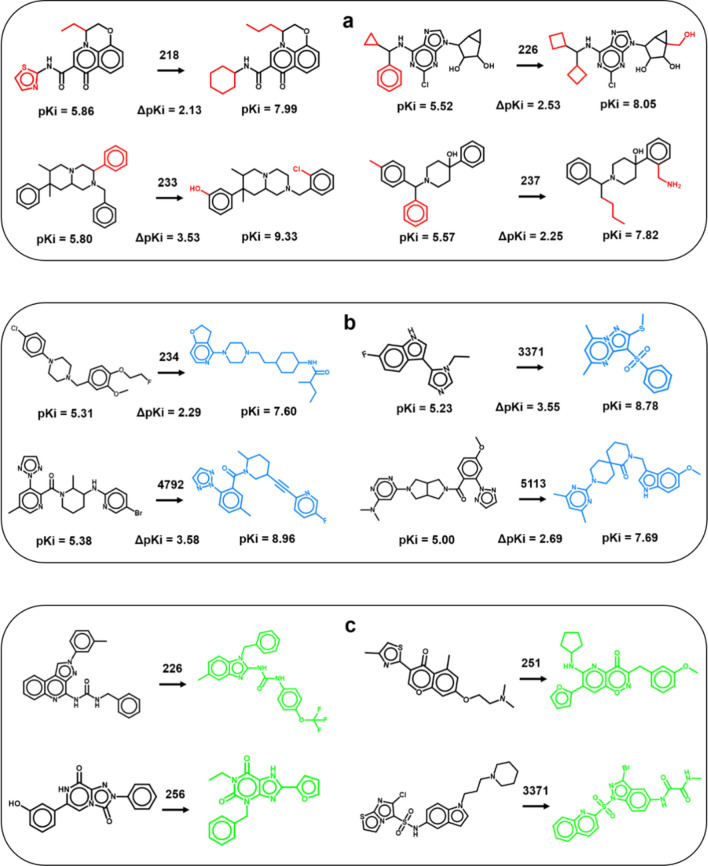
Exemplary predictions. Shown are pairs of corresponding source compounds (left of the arrow) and new compounds generated by the CLM (right) including (**a**) potent known compounds with conserved core structures (black, distinguishing substituents are red), (**b**) potent known compounds with distinct structures (blue), and (**c**) hypothetical compounds (green). For hypothetical compounds, no potency values were available. Numbers on arrows identify activity classes according to Table 1. Potency differences between SCs and KCCs are reported.

Figure 4 shows exemplary pairs of SCs and newly designed compounds (TCs) with different structural relationships. Given our design strategy, all SCs were known compounds with experimentally determined potency. The generated TCs included known potent analogues of SCs (Fig. 4a), structurally distinct known potent compounds (Fig. 4b), and novel (hypothetical) compounds (Fig. 4c). Taken together, these examples illustrate successful CLM predictions.

## Conclusion

The underlying idea for the development of the approach reported herein was to predict highly potent compounds from individual weakly potent input molecules. For all practical purposes, this represents an ultimate goal of potency prediction, especially for compound optimization in medicinal chemistry. This prediction task could not be addressed using conventional regression models. In addition, going beyond the applicability domain of standard QSAR modeling, we also aimed to design structurally diverse compounds, in addition to analogues. Therefore, a different methodological framework was required and we adapted a conditional transformer architecture previously used for AC predictions. These predictions established that compound generation could be conditioned on potency differences. However, since AC predictions were also confined to structurally analogous compounds, it remained unclear whether or not potency difference conditioning was transferable to the design of structurally diverse compounds with high potency. The CLM reported herein was fine-tuned on pairs of SCs and TCs with associated potency differences and we then examined its ability to predict structurally diverse compounds with large increases in potency relative to input molecules. Therefore, a compound pair-based test system was generated that covered all possible prediction outcomes and enabled a well-defined and rigorous assessment of model performance. Our analysis confirmed the ability of the model to reproduce known potent compounds not encountered during training at unexpectedly high rates, including both analogues of weakly potent SCs and structurally distinct compounds. With median potency increases close to or above 100-fold across activity classes and multiple predictions with more than 1000-fold increases in compound potency, model performance was generally high. In addition, the CLM also produced large numbers of novel compounds for the activity classes that were not contained in the fine-tuning or test set.

Taken together, our findings indicate that the approach reported herein should have considerable potential for practical applications. In compound optimization, we envision that the CLM will be fine-tuned using sets of active compounds for a target of interest and that the predictions will then focus on input compounds prioritized by medicinal chemistry. For these and other applications, the CLM is made freely available as a part of our study.

## Data Availability

All calculations were carried out using publicly available programs and compound data. Python scripts used for implementing CLMs and the activity classes used herein are freely available via the following link: https://doi.org/10.5281/zenodo.7744763.
